# Knowledge of Infectious Disease Specialists Regarding Aspergillosis Complicating Influenza, United States

**DOI:** 10.3201/eid2604.190953

**Published:** 2020-04

**Authors:** Mitsuru Toda, Susan E. Beekmann, Philip M. Polgreen, Tom M. Chiller, Brendan R. Jackson, Karlyn D. Beer

**Affiliations:** Centers for Disease Control and Prevention, Atlanta, Georgia, USA (M. Toda, T.M. Chiller, B.R. Jackson, K.D. Beer);; University of Iowa, Iowa City, Iowa, USA (S.E. Beekmann, P.M. Polgreen)

**Keywords:** knowledge, infectious disease specialists, invasive pulmonary aspergillosis, fungi, viruses, influenza virus, influenza, severe influenza, galactomannan, United States

## Abstract

In an online survey, we found that nearly one fifth of physicians in the United States who responded had seen or heard about a case of invasive pulmonary aspergillosis after severe influenza at their institution. However, <10% routinely used galactomannan testing to test for this fungus in patients with severe influenza.

Invasive pulmonary aspergillosis (IPA) occurs primarily among immunocompromised patients with a history of organ or stem cell transplantation, chemotherapy, or immunosuppressive medications. However, a multicenter retrospective study in the Netherlands and Belgium suggested that patients with severe influenza (i.e., requiring intensive care unit [ICU] admission) are also at risk for IPA (*1*). In that study, 19% patients with severe influenza showed development of IPA. More than half of these patients were not immunocompromised, and mortality rates were twice as high among ICU patients with IPA compared with those without IPA.

Corticosteroids, which have been associated with higher mortality rates and are used for influenza patients (*2*), are a known risk factor for IPA and have been associated with IPA in severe influenza (*3*). However, 44% of patients who showed development of IPA in the study in the Netherlands and Belgium had not received these medications (*1*). Although case reports exist (*4*,*5*), clinicians might not consider IPA as a cause of worsening respiratory function or sepsis because influenza is not considered a classical risk factor for IPA and because of the complexity inherent in diagnosis (*6*). In the study in the Netherlands and Belgium, IPA cases were diagnosed by galactomannan antigen testing of bronchoalveolar lavage fluid (*1*). Although galactomannan testing might be useful in the ICU setting (*7*), it is unclear how often galactomannan testing is performed in the United States.

To clarify clinical practices regarding diagnosis of IPA in patients with severe influenza, the Emerging Infections Network (EIN) surveyed infectious disease specialists in the United States. EIN is a provider-based emerging infections sentinel network supported by the Centers for Disease Control and Prevention and the Infectious Diseases Society of America (*8*). During May‒June 2018, EIN distributed a 6-question poll to its >1,500 member listserv (https://ein.idsociety.org); 114 responded.

Twenty-nine (26%) respondents were familiar with reports of aspergillosis after severe influenza, and 21 (18%) had seen or heard about >1 case at their institution (Table). Among 108 responding clinicians, 33 (31%) always or very often used lower respiratory tract specimens for diagnostic testing in patients with severe influenza. Only 8 (8%) of 107 clinicians always or very often used lower respiratory specimens and galactomannan testing in patients with severe influenza in the ICU and worsening respiratory function.

**Table:** Summary results of survey on invasive pulmonary aspergillosis accompanying severe influenza among a network of infectious disease specialists, United States, May–June, 2018*

Survey characteristic	No. (%)
Region where respondents are from, n = 114	
Midwest	25 (22)
Northeast	27 (24)
South	33 (29)
West	29 (25)
Familiar with reports of aspergillosis after severe influenza infection, n = 114	
Yes, familiar with reports	29 (26)
No, not familiar with reports	83 (73)
Seen or heard about a case of aspergillosis in the setting of severe influenza at place of work, n = 114	
Yes, 1 case	15 (13)
Yes, >2 cases	6 (5)
No	93 (82)
For patients with influenza requiring ICU admission, how commonly are lower respiratory specimens (e.g., bronchoalveolar lavage, bronchial wash) obtained, n = 108	
Never	2 (2)
Rarely	28 (26)
Sometimes	45 (42)
Very often	28 (26)
Always	5 (5)
When treating patients with severe influenza in the ICU and worsening respiratory function, how often do you order galactomannan testing (e.g., in serum or bronchoalveolar lavage), n = 107	
Never	30 (28)
Rarely	45 (42)
Sometimes	24 (22)
Very often	4 (4)
Always	4 (4)

*n values indicate number of participants who responded. ICU, intensive care unit.

Most respondents were unaware of concerns about IPA in severe influenza, suggesting that physicians might not consider it in their differential diagnosis. In addition, most respondents reported infrequent use of galactomannan testing in patients with severe influenza, which might limit ability to detect IPA.

Although our response rate and possible selection bias might limit our ability to draw conclusions, ≈20% of respondents had seen or heard about an IPA case at their institution. IPA in patients with severe influenza might be more common than appreciated based on small numbers of previously published cases in the United States (*4*,*5*). Additional research and surveillance are needed to understand the association between IPA and severe influenza and performance of galactomannan testing in patient with severe influenza. Nonetheless, it is essential for clinicians to consider IPA in patients with severe influenza who do not improve with treatment, even in those who are not immunocompromised.
